# Tai Chi for cardiovascular wellness: Integrating an ancient practice into modern therapeutic approaches

**DOI:** 10.37796/2211-8039.1678

**Published:** 2025-12-01

**Authors:** Chun-Han Cheng, Wen-Rui Hao, Huan-Yuan Chen, Po-Yuan Chen, Ju-Chi Liu, Tzu-Hurng Cheng

**Affiliations:** a Department of Medical Education, Linkou Chang Gung Memorial Hospital, Taoyuan City 33305, Taiwan, ROC; b Division of Cardiology, Department of Internal Medicine, Shuang Ho Hospital, Ministry of Health and Welfare, Taipei Medical University, New Taipei City 23561, Taiwan, ROC; c Division of Cardiology, Department of Internal Medicine, School of Medicine, College of Medicine, Taipei Medical University, Taipei City 11002, Taiwan, ROC; d Institute of Biomedical Sciences, Academia Sinica, Taipei City 115201, Taiwan, ROC; e Department of Biological Science and Technology, College of Life Sciences, China Medical University, Taichung City 404333, Taiwan, ROC; f Department of Biochemistry, School of Medicine, College of Medicine, China Medical University, Taichung City 404333, Taiwan, ROC

**Keywords:** Tai Chi, Cardiovascular health, Cardiovascular rehabilitation, Complementary therapy, Stress reduction, Exercise therapy

## Abstract

Tai Chi, a traditional Chinese martial art characterized by gentle, fluid movements and deep breathing, has gained increasing recognition for its cardiovascular health benefits. This study investigated the integration of Tai Chi into contemporary cardiovascular health practices, focusing on its physiological and psychological effects. The slow, controlled movements characteristic of Tai Chi contribute to enhanced cardiovascular fitness, decreased blood pressure, and improved vascular function, while simultaneously alleviating stress and fostering emotional well-being. Through a review of clinical studies and trials, this study underscores the efficacy of Tai Chi in cardiovascular rehabilitation programs and its accessibility as a community-based intervention. Additionally, this study addresses obstacles to widespread adoption, including cultural barriers and the lack of standardized training for instructors. By integrating traditional practices with contemporary medical approaches, Tai Chi is as a valuable complementary therapy for cardiovascular health. The paper presents future research directions and advocacy strategies aimed at promoting broader acceptance and implementation of Tai Chi in health-care settings. This review underscores the continued relevance of Tai Chi as an effective intervention for cardiovascular wellness in modern therapeutic contexts.

## 1. Introduction

Tai Chi, an ancient Chinese practice combining slow, meditative movements with mindful breathing, has evolved significantly from its origins in martial arts to become a globally recognized exercise for health and wellness [1,2].

Initially developed as a martial art, Tai Chi’s deliberate, flowing movements are designed to cultivate internal energy, or “Qi (Chi),” while fostering physical and mental equilibrium. Over centuries, it has been celebrated for improving not only physical fitness but also mental well-being and overall life balance [2,3]. Today, Tai Chi is widely acknowledged as a holistic practice that integrates the body, mind, and spirit, offering a variety of health benefits, particularly in the context of cardiovascular health [4]. Cardiovascular diseases (CVDs), including coronary artery disease, hypertension, and heart failure, remain the leading causes of death globally, significantly contributing to morbidity and mortality [3,5]. Traditional approaches to cardiovascular health emphasize lifestyle interventions such as diet and regular exercise, along with pharmacological treatments. However, the rising prevalence of CVDs has spurred interest in complementary therapies like Tai Chi, which can enhance conventional treatments and offer a more holistic approach to care [4,6,7]. Integrating Tai Chi into cardiovascular health management reflects a blend of ancient wisdom with modern medical practices. This low-impact, accessible form of exercise is particularly suited to individuals with cardiovascular conditions, offering a gentle yet effective means of improving cardiovascular function without undue physical strain [4,8]. Studies suggest Tai Chi enhances cardiovascular fitness, lowers blood pressure, and positively impacts lipid profiles, all of which are crucial in managing CVDs [4,9,10].

**Fig. 1:**
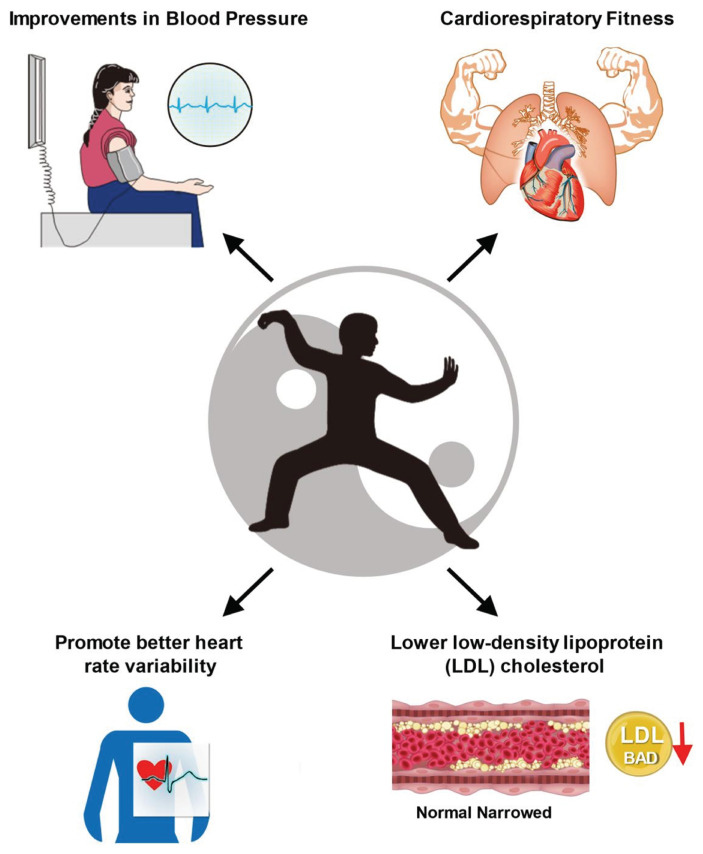
Impact of Tai Chi on cardiovascular health: improvements in blood pressure, cardiorespiratory fitness, and vascular function. This figure illustrates the physiological benefits of Tai Chi for cardiovascular health. Key improvements include the reduction of systolic and diastolic blood pressure, enhanced cardiorespiratory fitness through moderate aerobic activity, and improved vascular function as evidenced by increased endothelial flexibility and arterial compliance. Studies also highlight Tai Chi’s ability to lower low-density lipoprotein (LDL) cholesterol, improve lipid profiles, and promote better heart rate variability. Together, these benefits contribute to overall cardiovascular wellness and reduce risk of cardiovascular diseases.

Also, Tai Chi’s mindfulness and meditative elements play a crucial role in reducing stress, a significant risk factor for cardiovascular diseases [11,12]. By promoting relaxation and emotional balance, Tai Chi offers a comprehensive approach to cardiovascular health (see Fig. 1). The purpose of this article is to provide a narrative review of current research on Tai Chi’s potential benefits for cardiovascular health. While it does not employ a systematic review or meta-analysis, this article synthesizes existing studies, highlights key findings, and outlines Tai Chi’s role in cardiovascular care. Practical considerations for incorporating Tai Chi into cardiovascular rehabilitation programs are discussed, along with challenges to broader adoption, such as cultural perceptions and the need for standardized instructor training [13]. Although Tai Chi shows great promise as a complementary therapy, further research, including systematic reviews and meta-analyses, is essential to firmly establish its role in evidence-based cardiovascular care [4,14]. Briefly, this paper advocates for the broader acceptance of Tai Chi in cardiovascular health management, emphasizing its potential to bridge traditional and modern healthcare practices. By providing a balanced, integrative approach, Tai Chi aligns with patients’ holistic needs and offers a valuable addition to contemporary cardiovascular wellness strategies [4,8,15].

## 2. Understanding Tai Chi

Tai Chi, or Tai Chi Chuan, is a widely practiced, low-impact exercise renowned for its health benefits. Rooted in ancient Chinese philosophy, Tai Chi embodies the principles of Yin and Yang, symbolizing balance and harmony. This practice involves a series of slow, deliberate movements synchronized with controlled breathing and focused mental attention, promoting both physical and mental well-being [1]. At the core of Tai Chi is the concept of Qi, the vital energy believed in traditional Chinese medicine (TCM) to flow throughout the body, essential for maintaining health. Tai Chi aims to enhance the circulation of Qi through the body’s meridian system, increasing vitality and reducing stress [2]. Tai Chi encompasses several distinct styles, each with unique characteristics but sharing the same foundational principles. The most popular styles are Chen, Yang, Wu, and Sun. Chen, the oldest style, is known for its dynamic movements and low stances. The Yang style, the most widely practiced, features gentle, flowing movements, making it accessible to people of all ages and fitness levels. The Wu style is characterized by smaller, more compact movements, while the Sun style, which incorporates elements of Tai Chi, Xingyiquan, and Baguazhang, is recognized for its agile, smooth motions [3]. Each style of Tai Chi includes specific forms or sets―sequences of movements performed slowly and continuously. These forms vary in complexity and duration, from brief routines to longer, intricate sequences. Regardless of style or form, Tai Chi’s core principles―relaxation, proper alignment, and the integration of body and mind―remain consistent [6]. Beyond physical exercise, Tai Chi is a holistic mind-body practice. By coordinating breath with movement and focusing the mind, Tai Chi becomes a meditative discipline that promotes mental clarity and emotional stability. Many practitioners report feeling calm and centered after practice, attributing these effects to Tai Chi’s meditative qualities [4].

Increasing scientific evidence supports Tai Chi’s health benefits, particularly in reducing stress, improving balance and flexibility, and supporting cardiovascular health (see Table 1). Tai Chi’s relaxation and stress-mitigation benefits help lower blood pressure and improve heart rate variability―key factors in cardiovascular health [8]. In sum, Tai Chi is a multifaceted practice rooted in ancient Chinese philosophy that combines physical movement, breath control, and mental focus to foster holistic well-being. Its diverse styles and forms make it accessible to a wide audience, while its integrative approach offers substantial benefits for physical, mental, and emotional health [13,14].

## 3. Cardiovascular health and traditional therapies

Cardiovascular health is essential for overall wellbeing, as it relies on the efficient functioning of the heart and blood vessels to maintain proper blood flow and nutrient delivery throughout the body [3]. Traditional management of cardiovascular health typically includes pharmacological treatments, lifestyle changes such as diet and exercise, and, in some cases, surgical interventions. Despite these approaches, the growing prevalence of cardiovascular diseases (CVDs) has spurred interest in complementary and alternative therapies to enhance conventional treatments and support holistic care [16]. Traditional therapies encompass a diverse range of practices rooted in cultural and historical traditions, including TCM, Ayurveda, and other indigenous medical systems [6]. These approaches prioritize balancing bodily systems, promoting holistic health, and emphasizing preventive care. In cardiovascular health, therapies like acupuncture, herbal medicine, and mindbody practices such as Tai Chi and Qigong are gaining attention for their potential benefits [2]. Tai Chi, known for its gentle, flowing movements combined with focused breathing and mindfulness, has emerged as a particularly promising therapy for cardiovascular health. Unlike high-intensity exercises that may not be suitable for individuals with cardiovascular conditions, Tai Chi provides a low-impact, accessible form of physical activity that can enhance cardiovascular fitness without placing excessive strain on the heart [3]. Regular practice of Tai Chi has been linked to reductions in blood pressure, improvements in lipid profiles, and overall enhanced cardiovascular function [17]. Integrating Tai Chi and other traditional therapies into cardiovascular care represents a shift toward an integrative health approach, blending modern medical practices with ancient traditions to address the complexities of cardiovascular health. Tai Chi, by promoting relaxation and reducing stress, helps mitigate key risk factors for CVDs [18]. Its meditative qualities also support emotional well-being, which is increasingly recognized as an important factor in cardiovascular health [9]. Clinical research reinforces the efficacy of traditional therapies like Tai Chi, with studies showing notable improvements in cardiovascular markers and quality of life for those who incorporate it into their routines [8]. These findings suggest that traditional therapies can play a valuable role in comprehensive cardiovascular care, particularly for individuals seeking non-pharmacological interventions [8]. In brief, integrating traditional therapies such as Tai Chi into cardiovascular health practices offers a holistic approach that complements conventional treatments. By bridging ancient wisdom with modern medicine, these practices provide a balanced strategy for managing cardiovascular health, addressing both the physical and psychological aspects of wellbeing [1,19].

## 4. Mechanisms of Tai Chi’s cardiovascular benefits

**Fig. 2:**
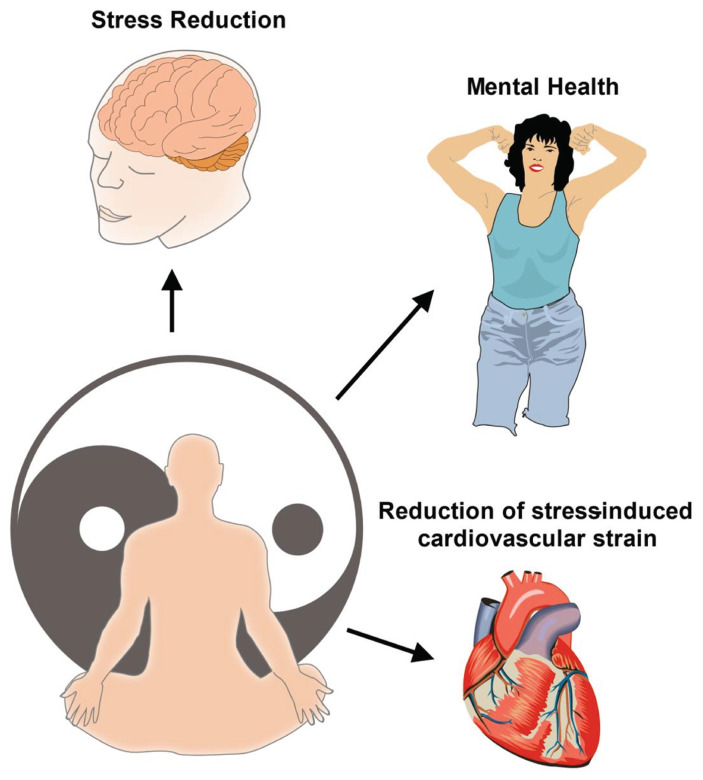
Tai Chi as a complementary therapy: enhancing mental health and stress reduction in cardiovascular rehabilitation. This figure demonstrates the psychological benefits of Tai Chi as a complementary therapy in cardiovascular rehabilitation. Tai Chi’s meditative movements and focus on controlled breathing help reduce stress and anxiety, leading to lower cortisol levels, improved emotional balance, and enhanced psychological resilience. These mental health improvements, along with the reduction of stress-induced cardiovascular strain, contribute to better overall rehabilitation outcomes and quality of life for patients with cardiovascular conditions.

The cardiovascular benefits of Tai Chi arise from its unique blend of physical activity, stress reduction, and mind-body integration. Understanding these mechanisms involves examining both the physiological and psychological impacts of this ancient practice. Tai Chi’s slow, deliberate movements contribute to cardiovascular fitness through moderate aerobic activity, raising the heart rate and promoting blood circulation without excessive strain. Studies show that regular practice improves endothelial function, which is essential for vascular health. Endothelial cells lining blood vessels play a crucial role in regulating blood pressure and preventing atherosclerosis; by supporting healthy endothelial function, Tai Chi helps maintain flexible, healthy blood vessels, reducing the risk of hypertension and other cardiovascular diseases [3]. Moreover, Tai Chi positively impacts lipid profiles by lowering low-density lipoprotein (LDL) cholesterol and triglycerides while increasing high-density lipoprotein (HDL) cholesterol, which reduces the risk of plaque buildup in the arteries, a major factor in coronary artery disease [6]. The practice also stimulates the lymphatic system, promoting detoxification and supporting overall cardiovascular health [4]. Research further underscores Tai Chi’s role in improving heart rate variability, which reflects better autonomic regulation and cardiovascular function. For instance, combining Tai Chi with resistance band exercises has been shown to enhance heart rate variability and overall fitness in older adults [4,8]. Improved heart rate variability is particularly beneficial in reducing stress and promoting heart health. Systematic reviews and meta-analyses confirm that Tai Chi significantly enhances cardiorespiratory fitness, increasing oxygen uptake and supporting efficient heart and lung function [13]. These physiological improvements collectively contribute to a reduced risk of cardiovascular disease and better cardiovascular well-being. Stress is a significant risk factor for cardiovascular disease, contributing to conditions such as hypertension, heart attacks, and strokes. With its emphasis on controlled breathing and mental focus, Tai Chi serves as an effective tool for managing stress. By promoting mindfulness and present-moment awareness, Tai Chi reduces cortisol levels―a hormone closely associated with stress [14]. Lower cortisol levels, in turn, help reduce blood pressure and heart rate, easing strain on the cardiovascular system. Tai Chi also enhances psychological resilience by improving heart rate variability, an indicator of a healthier autonomic nervous system and cardiovascular function [8]. Elevated heart rate variability not only improves the body’s stress response but also promotes cardiovascular stability during challenging situations [20]. These psychological benefits, including reduced stress and enhanced heart rate variability, are central to Tai Chi’s contributions to cardiovascular health. Clinical studies further validate Tai Chi’s cardiovascular benefits. Regular practitioners show significant improvements in cardiovascular markers, with lower blood pressure and improved arterial compliance compared to non-practitioners [16]. Tai Chi also reduces levels of C-reactive protein, an inflammatory marker linked to cardiovascular risk [17]. This moderate aerobic exercise enhances cardiorespiratory fitness [13], while its effects on endothelial function support vascular health, reducing the risk of hypertension and atherosclerosis [18]. Additionally, Tai Chi improves lipid profiles by lowering LDL cholesterol and triglycerides and increasing HDL cholesterol, which helps prevent arterial plaque buildup [9]. Furthermore, Tai Chi promotes better heart rate variability, aiding in stress regulation and cardiovascular stability, particularly beneficial for those with chronic heart conditions. The practice also supports lymphatic system function, aiding detoxification and contributing to cardiovascular health [4]. Overall, Tai Chi offers a holistic approach to cardiovascular health by combining moderate aerobic exercise, enhanced endothelial function, improved lipid profiles, stress reduction, and increased heart rate variability (see Fig. 2). These combined effects make Tai Chi a powerful integrative practice that promotes both physical and mental well-being, bridging the gap between traditional exercise and mind-body wellness.

## 5. Case studies and practical applications

Case studies and practical applications highlight Tai Chi’s positive impact on cardiovascular health, showcasing its potential as a valuable addition to comprehensive care. Numerous case reports illustrate Tai Chi’s broad benefits across various demographics and health conditions. For example, research on older adults practicing abdominal breathing through Tai Chi showed notable improvements in heart rate variability, a key indicator of cardiovascular health [3]. Likewise, a study involving patients with advanced lung cancer found that Tai Chi improved sleep quality more effectively than other aerobic exercises [6]. In addition, a systematic review identified Tai Chi as an effective component in alternative models of cardiac rehabilitation, reinforcing its role in promoting cardiovascular health [4]. Collectively, these examples underscore Tai Chi’s versatility and effectiveness in enhancing cardiovascular outcomes across different populations and conditions (see Table 2). Tai Chi’s gentle yet powerful approach has led to its integration into cardiac rehabilitation programs, providing patients with a holistic way to improve physical health while alleviating psychological stress. Many hospitals and rehabilitation centers now incorporate Tai Chi into their programs, reporting significant improvements in both physical fitness and mental wellbeing among participants [8]. Community-based Tai Chi classes have also expanded access, with local health organizations and senior centers frequently offering tailored programs for older adults aimed at improving cardiovascular health, balance, and fall prevention [13]. These classes provide a supportive environment where participants can safely engage in beneficial physical activity, enhancing both cardiovascular health and overall well-being [14]. To maximize the cardiovascular benefits of Tai Chi, proper instructor training and certification are essential. Qualified instructors can adapt sessions to meet the needs of individuals with cardiovascular conditions, ensuring both safety and effectiveness [8]. Guidelines recommend starting with shorter, less intense sessions and gradually increasing duration and complexity as fitness levels improve. Raising awareness of Tai Chi’s advantages through patient education and healthcare provider recommendations can also encourage broader adoption. Healthcare professionals should consider suggesting Tai Chi as a complementary therapy, especially for patients who would benefit from a low-impact, stress relieving exercise [13]. Integrating Tai Chi into cardiovascular care offers a holistic approach that supports both physical and mental health. Case studies and practical applications demonstrate its effectiveness in improving cardiovascular markers and enhancing overall well-being. By incorporating Tai Chi into rehabilitation programs and community classes, healthcare providers can offer a versatile, accessible tool for supporting cardiovascular health.

## 6. Challenges and considerations

Despite its numerous benefits, integrating Tai Chi into cardiovascular health practices presents several challenges that must be addressed to ensure safe and effective implementation. One significant barrier is the difficulty of establishing Tai Chi programs in certain environments, such as assisted living facilities. Constraints like limited space, a shortage of trained staff, and logistical issues can impede successful integration [3]. Also, cultural perceptions of Tai Chi as a traditional rather than a structured exercise regimen may deter individuals more accustomed to Western fitness methods [6]. Some may view Tai Chi as too low in intensity compared to activities like jogging or cycling, even though evidence demonstrates its positive effects on cardiovascular and overall health [4]. Overcoming these barriers requires a coordinated effort to educate healthcare providers and the public on the tangible benefits of Tai Chi and its practical applications across various healthcare settings. While generally safe for most individuals, Tai Chi still necessitates certain precautions, especially for specific populations. Such as, individuals with chronic heart failure may need careful monitoring and supervision during sessions to ensure safety and maximize benefits [8]. Pregnant women should also approach Tai Chi cautiously, as the effects of Tai Chi on pregnancy outcomes are not fully understood and modifications may be required to accommodate physiological changes [13]. Furthermore, people with musculoskeletal injuries or balance disorders should consult healthcare professionals to determine the suitability of Tai Chi and any necessary adjustments [14]. Personalized assessments and guidance are essential to ensure safe practice and minimize risks. Despite its proven benefits, access to Tai Chi programs remains limited for certain populations. Implementing Tai Chi in assisted living facilities has shown a reduction in fall risks and an improvement in quality of life for older adults [3]; however, disparities persist, particularly for marginalized communities. Ensuring inclusivity and equity in Tai Chi program availability is crucial, allowing individuals―regardless of socioeconomic status or location―to benefit from the practice. Partnerships between community organizations and healthcare providers can help overcome access barriers and extend Tai Chi programs to underserved populations. Addressing the challenges of integrating Tai Chi into cardiovascular health practices is essential to maximizing its benefits and ensuring wider access. By overcoming barriers to adoption, prioritizing safety, and promoting inclusivity, Tai Chi can become a valuable complementary therapy in modern cardiovascular care. Collaborative efforts among healthcare providers, policymakers, and community stakeholders are key to unlocking Tai Chi’s full potential for promoting cardiovascular health for all.

## 7. Future directions

With growing evidence supporting Tai Chi’s cardiovascular benefits, several promising pathways for future research and practice have emerged, offering opportunities for deeper integration and optimization within modern healthcare. Although Tai Chi’s advantages are increasingly recognized, key research gaps remain. Further studies are needed to explore its effects on specific health conditions, such as sleep quality in patients with advanced lung cancer [3], as well as its potential for managing insomnia and improving sleep health in broader populations [6]. Research should also examine Tai Chi’s efficacy among specific groups, like pregnant women, and assess its impact on physical, mental, and cognitive well-being across different age ranges [4,8]. Addressing these gaps will broaden our understanding of Tai Chi’s diverse applications and refine its use in clinical settings. A primary research focus should be on understanding Tai Chi’s benefits for special populations. For example, tailoring Tai Chi interventions for pregnant women could significantly enhance physical activity levels, supporting improved health outcomes [4]. Moreover, examining Tai Chi’s role in managing chronic conditions, such as heart failure, will offer insights into its safety and effectiveness for vulnerable populations [13]. Customizing interventions for diverse groups will optimize health outcomes and enhance quality of life for a wider range of individuals. Integrating Tai Chi into mainstream healthcare will require addressing institutional barriers and fostering acceptance among healthcare providers. Developing evidence-based guidelines for incorporating Tai Chi into cardiovascular rehabilitation programs will establish a framework for standardized practice [13,14]. Collaboration between healthcare providers, Tai Chi instructors, and community organizations can facilitate the implementation of Tai Chi initiatives within existing care pathways, ensuring patients benefit from this holistic approach [4,20]. Advocacy will be essential for expanding Tai Chi’s reach in healthcare. Raising awareness of its cardiovascular benefits among policymakers, healthcare organizations, and insurers can help drive policy changes, including reimbursement for Tai Chi programs [6]. Public education campaigns that highlight Tai Chi’s evidence-based benefits while dispelling misconceptions can also promote greater participation, particularly among individuals at risk for cardiovascular disease. The future of Tai Chi in cardiovascular health lies in advancing research, tailoring interventions for specific populations, integrating Tai Chi into healthcare systems, and advocating for its broader adoption. By addressing these priorities, Tai Chi has the potential to become a widely recognized therapy for promoting cardiovascular wellness and enhancing overall health and well-being.

## 8. Conclusion

Tai Chi presents a promising approach to cardiovascular wellness, skillfully merging ancient wisdom with contemporary medical practice. Through gentle movements, controlled breathing, and meditative focus, Tai Chi contributes notable cardiovascular benefits, such as improved fitness, blood pressure regulation, and reduced stress [21,22]. Clinical studies affirm Tai Chi’s effectiveness in enhancing cardiovascular markers and overall quality of life, establishing it as a valuable complementary therapy in cardiovascular care. While challenges remain in the widespread adoption of Tai Chi, ongoing efforts are focused on integrating it into mainstream healthcare systems, fueled by growing evidence of its efficacy and increasing patient demand for alternative therapies [23]. Overcoming barriers to access, ensuring safe practice, and fostering cultural competence are essential steps toward fully realizing Tai Chi’s potential for cardiovascular health. Future research should prioritize addressing existing knowledge gaps, tailoring interventions for specific populations, and advocating for policy changes that facilitate wider adoption [24,25]. By fostering collaboration among healthcare providers, policymakers, and community stakeholders, Tai Chi can become a mainstream therapy for cardiovascular wellness, empowering individuals to lead healthier, more balanced lives. With its accessible, gentle nature and profound mind-body benefits, Tai Chi is poised to transform cardiovascular care and advance holistic well-being.

## Figures and Tables

**Table 1:** Cardiovascular benefits of Tai Chi.

Benefit	Description	Reference
Blood Pressure Control	Tai Chi has been shown to lower both systolic and diastolic blood pressure in individuals with hyper-tension, enhancing overall cardiovascular health.	Robins et al., 2012 [1]; Kim & Thiruvengadam, 2024 [3]
Improved Cardiorespiratory Fitness	Regular Tai Chi practice improves aerobic capacity and respiratory function, which contributes to better endurance and reduces cardiovascular disease risk.	Galantino et al., 2013 [6]; Danilov & Frishman, 2023 [4]
Cholesterol Management	Tai Chi has been linked to improved lipid profiles, reducing LDL cholesterol and triglycerides while increasing HDL cholesterol.	Lewis [2]; Burke et al. [8]
Enhanced Vascular Function	Tai Chi promotes better endothelial function and arterial compliance, which are critical for maintaining healthy blood vessels and preventing atherosclerosis.	Zhao et al. [9]; Liu et al. [20]
Mental Health and Stress Reduction	The meditative nature of Tai Chi helps alleviate stress, anxiety, and depression, reducing their contribution to cardiovascular disease risk.	Chen et al. [13]; Kuang et al. [19]
Balance and Coordination	Practicing Tai Chi enhances balance and coordination, reducing fall risk, which indirectly supports cardiovascular health in older adults.	Hansell et al. [11]; Wei et al. [26]
Anti-inflammatory Effects	Regular Tai Chi practice lowers inflammatory markers, which are involved in the development of cardiovascular diseases.	Alperson [14]; Wen & Su [15]
Weight Management	Tai Chi promotes physical activity and boosts metabolism, contributing to healthier weight management and reducing obesity-related cardiovascular risks.	Wang et al. [27]; Liu et al. [20]

This table illustrates the various cardiovascular benefits of Tai Chi, highlighting its role in improving blood pressure control, enhancing cardiorespiratory fitness, and promoting cholesterol management. The table also emphasizes Tai Chi’s positive effects on vascular function, mental health, and balance. Moreover, it outlines the anti-inflammatory properties and weight management benefits associated with regular practice, making Tai Chi a valuable therapeutic intervention for cardiovascular health across diverse populations. Each benefit is supported by relevant studies that underscore the holistic impact of Tai Chi on physical and mental well-being.

**Table 2:** Overview of clinical studies investigating the impact of Tai Chi on cardiovascular health.

Study	Study type	Participants	Intervention	Outcomes	Findings
Takemura et al. (2024) [21]	Randomized clinical trial	Advanced lung cancer patients	Tai Chi vs. aerobic exercise	Sleep quality, cardio-respiratory fitness	Tai Chi improved sleep quality and cardiorespiratory fitness similarly to aerobic exercise
Woo et al. (2024) [28]	Randomized controlled trial	Older adults with pre- frailty	Tai Chi with resistance band	Heart rate variability, functional fitness	Improved heart rate variability and functional fitness
Jiao et al. (2023) [29]	Randomized controlled trial (protocol)	Patients with chronic heart failure	Home-based Tai Chi rehabilitation	Cardiovascular function, quality of life	Anticipated benefits include improved heart function and reduced symptoms
Kohn et al. (2023) [30]	Randomized clinical trial	Older adults with hypertension	Tai Chi vs. health education	Mental health, resilience	Tai Chi improved mental health and psychological resilience
Yin et al. (2023) [10]	Systematic review & metaanalysis	Patients with essential hypertension	Tai Chi intervention	Blood pressure reduction, improved cardio-respiratory fitness	Tai Chi significantly improved systolic and diastolic blood pressure, enhanced cardiorespiratory fitness
Wen et al. (2021) [15]	Randomized Trial	Middle-aged and elderly patients	Tai Chi vs. control group	Hypertension and hyperlipidemia prevention	Significant reductions in systolic blood pressure and cholesterol levels in Tai Chi group

This table provides a synthesis of key studies evaluating Tai Chi’s effects on cardiovascular health, showcasing diverse populations and outcomes. Findings consistently suggest that Tai Chi can improve cardiovascular parameters such as blood pressure, heart rate variability, and overall cardiovascular function, particularly among older adults and patients with chronic conditions. The studies indicate that Tai Chi offers benefits comparable to conventional aerobic exercise, with additional improvements in mental health and quality of life.

## References

[1] Robins JL Elswick RK McCain NL The story of the evolution of a unique tai chi form: origins, philosophy, and research J Holist Nurs 2012 30 134 46 10.1177/0898010111423427 22228833 PMC3762493

[2] Lewis DE T’ai chi ch’uan Complement Ther Nurs Midwifery 2000 6 204 6 10.1054/ctnm.2000.0472 11858304

[3] Kim JH Thiruvengadam R Hypertension in an ageing population: diagnosis, mechanisms, collateral health risks, treatments, and clinical challenges Ageing Res Rev 2024 98 102344 10.1016/j.arr.2023.102344 38768716

[4] Danilov A Frishman WH Complementary therapies: Tai Chi in the prevention and management of cardiovascular disease Cardiol Rev 2023 37395587 10.1097/CRD.0000000000000468 37395587

[5] Raoufinia R Rahimi HR Abbaszadeh M Gholoobi A Saburi E Fakoor F Personalized approaches to cardiovascular disease: insights into FDAapproved interventions and clinical pharmacogenetics Curr Pharm Des 2024 30 1667 80 10.2174/1381612829666230216102050 38738725

[6] Galantino ML Callens ML Cardena GJ Piela NL Mao JJ Tai chi for wellbeing of breast cancer survivors with aromatase inhibitor-associated arthralgias: a feasibility study Altern Ther Health Med 2013 19 38 44 24254037

[7] Bakris GL Weber MA Overview of the evolution of hypertension: from ancient Chinese emperors to today Hypertension 2024 81 717 26 10.1161/HYPERTENSIONAHA.124.19001 38507509

[8] Burke DT Al-Adawi S Lee YT Audette J Martial arts as sport and therapy J Sports Med Phys Fitness 2007 47 96 102 10.1007/s11332-007-0007-3 17369805

[9] Zhao W Ju H Zhu K Meta-analysis of the intervention effects of tai chi on fasting blood glucose, blood pressure, and triglyceride in middle-aged and elderly people Aging Male 2024 27 2282977 10.1080/13685538.2023.2282977 38259166

[10] Yin Y Yu Z Wang J Sun J Effects of the different Tai Chi exercise cycles on patients with essential hypertension: a systematic review and metaanalysis Front Cardiovasc Med 2023 10 1016629 10.3389/fcvm.2023.1016629 36937925 PMC10020615

[11] Hansell AK Olmstead R López Maya E Banijamali S Stress reduction for paid home care aides: a feasibility study of mindfulness meditation and Tai Chi interventions Home Health Care Serv Q 2023 42 328 46 10.1080/01621424.2023.12094984 37194733

[12] Wang AYL Chang YC Chen KH Loh CYY Potential application of modified mRNA in cardiac regeneration Cell Transplant 2024 33 9636897241248956 10.1177/09636897241248956 38715279 PMC11080755

[13] Chen S Tai Z Liu J Barriers, facilitators, and sustainers in Tai Ji Quan practice: a mixed-methods RE-AIM assessment of college students versus the general population J Phys Act Health 2023 20 239 49 10.1123/jpah.2023-0275 36746154

[14] Alperson SY Tai Chi philosophy and nursing epistemology ANS Adv Nurs Sci 2008 31 E1 15 10.1097/01.ANS.0000311523.92047.98 20531257

[15] Wen J Su M A randomized trial of Tai Chi on preventing hypertension and hyperlipidemia in middle-aged and elderly patients Int J Environ Res Public Health 2021 18 5480 10.3390/ijerph18105480 34065454 PMC8160700

[16] Ma X Jennings G “Hang the flesh off the bones”: cultivating an “ideal body” in Taijiquan and Neigong” Int J Environ Res Public Health 2021 18 4417 10.3390/ijerph18084417 33919260 PMC8122597

[17] Chung HW Tai CJ Chang P Su WL Chien LY The Effectiveness of a traditional Chinese medicine-based mobile health app for individuals with prediabetes: randomized controlled trial JMIR Mhealth Uhealth 2023 11 e41099 10.2196/41099 37338977 PMC10337399

[18] Lu DP Influence of I-ching (Yijing, or The Book of Changes) on Chinese medicine, philosophy and science Acupunct Electrother Res 2013 38 77 133 10.3727/036012913X13642043565598 23724698

[19] Kuang X Dong Y Song L Dong L Chao G Zhang X The effects of different types of Tai Chi exercise on anxiety and depression in older adults: a systematic review and network meta-analysis Front Public Health 2023 11 1295342 10.3389/fpubh.2023.1295342 38259770 PMC10800705

[20] Liu HH Nichols C Zhang H Understanding Yin-Yang philosophic concept behind Tai Chi practice Holist Nurs Pract 2023 37 E75 82 10.1097/HNP.0000000000000498 37595124

[21] Takemura N Cheung DST Fong DYT Lee AWM Lam TC Ho JC Effectiveness of aerobic exercise and Tai Chi interventions on sleep quality in patients with advanced lung cancer: a randomized clinical trial JAMA Oncol 2024 10 176 84 10.1001/jamaoncol.2023.3757 38060250 PMC10704344

[22] Zhang K Guo H Zhang X Yang H Yuan G Zhu Z Effects of aerobic exercise or Tai Chi Chuan interventions on problematic mobile phone use and the potential role of intestinal flora: a multi-arm randomized controlled trial J Psychiatr Res 2024 170 394 407 10.1016/j.jpsychires.2023.06.014 38218013

[23] Ma N Chau JPC Deng Y Choi KC Effects of a structured Tai Chi program on improving physical activity levels, exercise self-efficacy, and health outcomes among pregnant women: study protocol for a randomised controlled trial BMJ Open 2023 13 e065640 10.1136/bmjopen-2022065640 PMC9944291 36806130

[24] Zhao FY Xu P Kennedy GA Conduit R Zhang WJ Wang YM Identifying complementary and alternative medicine recommendations for insomnia treatment and care: a systematic review and critical assessment of comprehensive clinical practice guidelines Front Public Health 2023 11 1157419 10.3389/fpubh.2023.1157419 37397764 PMC10308125

[25] Lee SY Nyunt MSZ Gao Q Gwee X Chua DQL Yap KB Association of Tai Chi exercise with physical and neurocognitive functions, frailty, quality of life, and mortality in older adults: Singapore Longitudinal Ageing Study Age Ageing 2022 51 afac086 10.1093/ageing/afac086 35380607

[26] Wei GX Li YF Yue XL Ma X Chang YK Yi LY Tai Chi Chuan modulates heart rate variability during abdominal breathing in elderly adults Psych J 2016 5 69 77 10.1002/pchj.120 26377754

[27] Wang Y Miao X Viwattanakulvanid P Effects of a therapeutic lifestyle modification intervention on cardiometabolic health, sexual functioning, and health-related quality of life in perimenopausal Chinese women: protocol for a randomised controlled trial BMJ Open 2024 14 e082944 10.1136/bmjopen-2023-082944 PMC11029459 38626978

[28] Woo SC Chen MY Chen LK Liu CY Effectiveness of resistance band use in conjunction with Tai Chi among older adults with prefrailty to improve functional fitness, quality of life, and heart rate variability J Gerontol Nurs 2024 50 19 26 10.3928/00989134-20230817-05 38691121

[29] Jiao Q Meng C He H Li S Xu F Cui W Safety and effects of a home-based Tai Chi exercise rehabilitation program in patients with chronic heart failure: study protocol for a randomized controlled trial Front Cardiovasc Med 2023 10 1237539 10.3389/fcvm.2023.1237539 38094121 PMC10716196

[30] Kohn JN Lobo JD Troyer EA Wilson KL Ang G Walker AL Tai chi or health education for older adults with hypertension: effects on mental health and psychological resilience to COVID-19 Aging Ment Health 2023 27 496 504 10.1080/13607863.2022.2156148 35311437 PMC9489818

